# Interventional treatment of symptomatic giant hepatic hemangiomas: initial results of the use of a combined technique

**DOI:** 10.1590/0100-3984.2021.0044

**Published:** 2021

**Authors:** Thiago Franchi Nunes, Tiago Kojun Tibana, Reinaldo Morais Neto, Edson Marchiori

**Affiliations:** 1 Hospital Universitário Maria Aparecida Pedrossian da Universidade Federal de Mato Grosso do Sul (HUMAP-UFMS), Campo Grande, MS, Brazil.; 2 Universidade Federal do Rio de Janeiro (UFRJ), Rio de Janeiro, RJ, Brazil.

## INTRODUCTION

The proper treatment of hepatic hemangiomas is a controversial topic in the literature. Surgical resection has been recommended for the treatment of symptomatic giant hepatic hemangiomas (SGHHs) and is considered the gold standard at most centers^**(1)**^. However, minimally invasive techniques, including transarterial embolization, have shown acceptable efficacy with lower rates of post-procedure morbidity and mortality^**(2-5)**^.

In a study conducted in Brazil, Szejnfeld et al.^**(5)**^ demonstrated that transarterial embolization using a lipiodol-ethanol mixture was a safe, effective treatment for SGHH in a small sample of patients. In a recent meta-analysis evaluating the effectiveness of transarterial embolization in the treatment of SGHHs, Torkian et al.^**(6)**^ concluded that transarterial embolization with lipiodol in combination with bleomycin, pingyangmycin, or ethanol is a safe procedure and is associated with a significant reduction in the size of the hemangiomas, resulting in symptom relief.

To date, there have been no studies of the combination of transarterial embolization and percutaneous ethanol injection in the treatment of giant hepatic hemangiomas. The purpose of this case series was to present the results of a safe, novel technique for the treatment of SGHHs.

## PROCEDURE

This was a retrospective study of four cases of SGHH in female patients who were treated with the combination of transarterial embolization and percutaneous ethanol injection between 2017 and 2021. None of the patients had a history of abdominal surgery. All liver masses showed a pattern typical of hemangioma on contrast-enhanced computed tomography or magnetic resonance imaging. No biopsies were performed.

**Figure 1:**
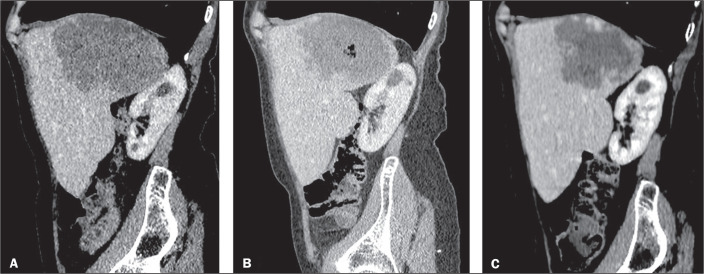
Contrast-enhanced abdominal computed tomography, with sagittal reconstruction, demonstrating a giant hemangioma. A: Volume estimated at 407 mL with signs of distension of the hepatic capsule on the diaphragm surface and compression of the upper pole of the right kidney. B: Follow-up examination performed 30 days after the procedure, showing a slight reduction in the dimensions and gaseous content of the hemangioma secondary to percutaneous sclerosis. C: Follow-up examination performed three months after the procedure, showing a reduction of approximately 50% in the size of the liver mass.

Embolization procedures were performed under conscious sedation. Initial diagnostic angiography was performed by using a 5F catheter to selectively examine the superior mesenteric artery, celiac trunk, and hepatic artery, including a late phase. A superselective technique using a 2.8F microcatheter (Progreat; Terumo Corporation, Tokyo, Japan) and 300-500 µm microparticles (Embospheres; BioSphere Medical Inc., Rockland, MA, USA) was employed to embolize the arterial branches supplying the tumor. Embolization was performed as selectively as possible, with very slow injection of the embolic agent, so that the endpoint was complete filling of the vascular sinusoids of the hemangioma with embolization material and iodinated contrast (Figure 1).

After the end of the arterial embolization procedure, percutaneous ethanol injection was performed by puncturing the center of the lesion with a 22G Chiba needle under real-time ultrasound guidance. In each procedure, 30 mL of absolute ethanol (100%) were injected. All patients received fentanyl (2 mL), ondansetron (8 mL), and a single dose of cefazolin (1 g).

Technical success was achieved in all four cases, and all of the patients were discharged from the hospital 24 h after the procedure, with only mild pain and no sign of complications. All of the patients also showed significant improvement in symptoms at the end of a three-month follow-up period. The results are summarized in Table 1.

## DISCUSSION

This case series demonstrates a combined technique that, to our knowledge, has not previously been reported. In their meta-analysis, Torkian et al.^**(6)**^ showed that transarterial embolization with bleomycin, pingyangmycin, or ethanol, in combination with lipiodol, is a safe procedure that is associated with a reduction in the size of hemangiomas, resulting in symptom relief. Clinical improvement was achieved in 100% of the cases in our sample, whereas Torkian et al.^**(6)**^ reported that the rate of clinical improvement ranged from 63% to 100%.

**Table 1:** Descriptions of four cases of SGHH treated with the combination of transarterial embolization and percutaneous ethanol injection.

Patient	Age (years)	Sex	SGHH location	SGHH size				SGHH volume reduction	Follow-up (months)
				Pre-treatment		Post-treatment (three months)			
				Maximum diameter (cm)	Volume (cm^3^)	Maximum diameter (cm)	Volume (cm^3^)		
1	65	Female	Right lobe	11	407	8	201	50.6%	10
2	42	Female	Right lobe	13.5	602	9.5	290	51.8%	15
3	35	Female	Left lobe	12.3	550	8.5	245	55.4%	19
4	40	Female	Right lobe	13.5	480	9.1	264	45.0%	25

## CONCLUSION

The combination of transarterial embolization and percutaneous ethanol injection is a technique that is safe, reproducible, and easy to perform. The use of this combination appears to result in significant symptom reduction and improvement in patients with unresectable SGHHs.
